# Supplementary material for: Posting patterns in peer online support forums and their associations with emotions and mood in bipolar disorder: Exploratory analysis
Source: PLoS One. 2023 Sep 25;18(9):e0291369. doi: 10.1371/journal.pone.0291369 (PMC10519601; doi:10.1371/journal.pone.0291369)
Supplement: S1 Table — The number of non-MH subreddits is the number of unique subreddits in which users in the dataset posted that are not in the MH subreddit list. The users in the dataset only posted in 36 of the 37 pre-identified BD-specific subreddits and in 116 of the 121 (158–37) not BD-specific subreddits in the MH subreddit list. (DOCX) [file pone.0291369.s001.docx]

S1 Table. Top 10 subreddits with most posts in the dataset for subreddits in the BD or MH subreddit list (excl. BD) and subreddits not in the MH subreddit list.

| BD subreddits (n=36) | | MH subreddits (excl. BD) (n=116) | | Non-MH subreddits (n=49,195) | |
| --- | --- | --- | --- | --- | --- |
| Top 10 subreddits | % of all 564,160 BD sub-reddit posts | Top 10 subreddits | % of all 618,165 MH sub-reddit posts | Top 10 subreddits | % of all 20,225,268 non-MH subred-dit posts |
| bipolar | 70.39 | stopdrinking | 13.04 | AskReddit | 14.49 |
| BipolarReddit | 24.66 | depression | 11.82 | politics | 1.98 |
| bipolar2 | 4.10 | raisedbynarcissists | 11.37 | funny | 1.69 |
| BipolarSOs | 0.53 | ADHD | 8.57 | pics | 1.56 |
| bipolarart | 0.08 | BPD | 8.26 | Random_Acts_Of_Amazon | 1.41 |
| cyclothymia | 0.07 | SuicideWatch | 4.39 | todayilearned | 1.09 |
| bipolar_irl | 0.05 | aspergers | 4.39 | AdviceAnimals | 1.05 |
| bipolarpoets | 0.02 | Anxiety | 4.17 | WTF | 1.05 |
| bipolar_r4r | 0.02 | cripplingalcoholism | 3.49 | AskWomen | 0.93 |
| BipolarCreativity | 0.01 | proED | 2.82 | news | 0.92 |

The number of non-MH subreddits is the number of unique subreddits in which users in the dataset posted that are not in the MH subreddit list. The users in the dataset only posted in 36 of the 37 pre-identified BD-specific subreddits and in 116 of the 121 (158-37) not BD-specific subreddits in the MH subreddit list.
