# Supplementary material for: Posting patterns in peer online support forums and their associations with emotions and mood in bipolar disorder: Exploratory analysis
Source: PLoS One. 2023 Sep 25;18(9):e0291369. doi: 10.1371/journal.pone.0291369 (PMC10519601; doi:10.1371/journal.pone.0291369)
Supplement: S2 Table — (DOCX) [file pone.0291369.s002.docx]

S2 Table. Descriptive statistics and between user differences for users in the regression model.

|  | Posted in MH subreddits | | Never posted in MH subreddits | | a: Independent t-test; b: Welch’s t-test | | |
| --- | --- | --- | --- | --- | --- | --- | --- |
|  | mean | std | mean | std | *P* | effect size a: Cohen's d  b: Glass’ delta | effect size interpre-tation [1] |
| Age | 29.22 | 9.31 | 29.47 | 9.82 | 2.28^a^ | -0.03^a^ | Very small |
| gender | 0.4 | 0.49 | 0.61 | 0.49 | <.001^a^ | -0.43^a^ | Small |
| active days | 1292.08 | 883.97 | 1130.44 | 889.61 | <.001^a^ | 0.18^a^ | Very small |
| activity | 1.44 | 2.52 | 1.85 | 4.79 | <.001^b^ | -0.16^b^ | Very small |
| posemo | 3.63 | 0.88 | 5.61 | 2.35 | <.001^b^ | 0.17^b^ | Very small |
| anxiety | 0.36 | 0.19 | 0.3 | 0.16 | <.001^a^ | 0.16^a^ | Very small |
| anger | 0.79 | 0.41 | 1.09 | 0.62 | <.001^a^ | -0.15^a^ | Very small |
| sadness | 0.44 | 0.21 | 0.43 | 0.22 | <.001^b^ | 0.14^b^ | Very small |
| 1^st^ pers. sg. | 6.35 | 1.86 | 5.54 | 1.75 | <.001^a^ | 0.21^a^ | Small |

### **References**

1. Sawilowsky SS. New Effect Size Rules of Thumb. J Mod Appl Stat Methods. 2009;8: 597–599. doi:10.22237/jmasm/1257035100
