# Supplementary material for: Posting patterns in peer online support forums and their associations with emotions and mood in bipolar disorder: Exploratory analysis
Source: PLoS One. 2023 Sep 25;18(9):e0291369. doi: 10.1371/journal.pone.0291369 (PMC10519601; doi:10.1371/journal.pone.0291369)
Supplement: S1 Appendix — (DOCX) [file pone.0291369.s003.docx]

S1 Appendix. Details on the creation of the BD and MH subreddit lists.

The following steps led to the list of 158 MH subreddits, of which 37 are specific to BD:

1. Starting point: three-level hierarchical topic-categorisation of 30K subreddits by snoopsnoo.com [1]: 20 first-, 141 second-, 529 third-level topics; snoopsnoo.com manually categorised subreddits based on subreddit descriptions and posts. Health and computational linguistics research [e.g., 2,3] has used this taxonomy previously.
2. The MH subreddit list was manually compiled from subreddits categorised in Lifestyle/Health, Lifestyle/Health/Depression and Anxiety, and Lifestyle/Health/Recovery.
3. MH-related subreddits from the SMHD dataset [4] were added to this list.
4. Subreddits from the MH list were categorised as BD-specific if according to their description they exclusively focused on BD.
5. Subreddits in which people with a self-reported BD diagnosis had posted that contained *bipolar* in their name, had more than 10K posts, or contained a BD diagnosis post or a post with a BD mention were added to the BD or MH subreddit list if appropriate.

### **References**

1. Orionmelt. Subreddit categorization. [cited 15 Aug 2022]. Available: https://github.com/orionmelt/sherlock/blob/master/subreddits.csv

2. Buntinx-Krieg T, Caravaglio J, Domozych R, Dellavalle RP. Dermatology on reddit: Elucidating trends in dermatologic communications on the world wide web. Dermatol Online J. 2017;23: 0–6.

3. Al-Khatib K, Völske M, Syed S, Kolyada N, Stein B. Exploiting Personal Characteristics of Debaters for Predicting Persuasiveness. Proceedings of the 58th Annual Meeting of the Association for Computational Linguistics. 2020. pp. 7067–7072.

4. Cohan A, Desmet B, Yates A, Soldaini L, MacAvaney S, Goharian N. MH subreddits. [cited 15 Aug 2022]. Available: https://ir.cs.georgetown.edu/data/smhd/mh_subreddits.txt
