# Supplementary material for: Posting patterns in peer online support forums and their associations with emotions and mood in bipolar disorder: Exploratory analysis
Source: PLoS One. 2023 Sep 25;18(9):e0291369. doi: 10.1371/journal.pone.0291369 (PMC10519601; doi:10.1371/journal.pone.0291369)
Supplement: S2 Appendix — (DOCX) [file pone.0291369.s004.docx]

S2 Appendix. Method details for the logistic regression model with controls and LIWC variables.

## Rationale for **model** selection

A logistic regression model was most appropriate to address RQ3 because it reveals which predictors significantly impact on the outcome and their effect sizes. While more complex models may yield higher accuracy, they are more difficult to interpret and therefore not suitable to address RQ3.

## Model estimation

The logistic regression model was fitted via the R [1, Windows version 4.1.0] glm function with the following specification:

model.liwc_w_controls <- glm(posted in MH ~ age + gender + active_days + activity + posemo + anxiety + anger + sadness + first_pers_sg, data = df, family = binomial(link="logit"))

This corresponds to describing the probability of a user having posted in MH subreddits via the following equation in vector notation:

$$p\left( \text{posted in MH} \right)=\frac{1}{1+e^{-(\beta_{0}+\beta_{1}\text{age+}\beta_{2}\text{gender+}\beta_{3}\text{active\_days+}\beta_{4}\text{activity+}\beta_{5}\text{posemo+}\beta_{6}\text{anxiety+}\beta_{7}\text{anger+}\beta_{8}\text{sadness+}\beta_{9}\text{first\_pers\_sg})}}$$

The model parameters $\beta_{0}$ (intercept) and predictor coefficients $\beta_{1}-\beta_{9}$ are fitted via maximum likelihood estimation.

The model was estimated using all 10,158 users which met the eligibility criteria and for which age and gender information was available. The outcome variable *posted in MH* was imbalanced with 7,846 (77.2%) of users having posted in MH subreddits (posted in MH = 1) and 2,312 (22.8%) of users only having posted in non-MH subreddits (posted in MH = 0). As S1 Fig. shows, the fitted model outperforms the majority class baseline that always predicts posted in MH = 1 for every user in the dataset for all recall levels except 1.0. Balancing the outcome variable by randomly subsampling 2,312 users with posted in MH = 1 only changed the intercept but not the predictor coefficients substantially, as expected.

The model was used to estimate the impact of each predictor on the outcome for the known users.


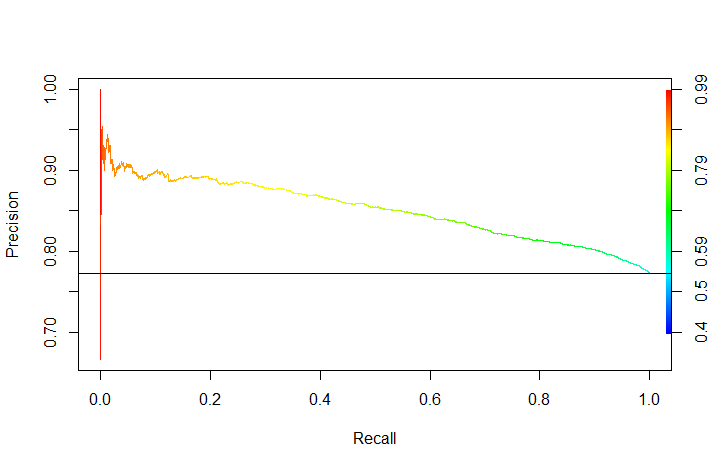


S1 Fig. Precision-recall plot for the glm regression model with controls and LIWC variables applied to the 10,158 users it was estimated on. Precision = TP / (TP + FP), Recall = TP (TP + FN), where True positives (TP) are users with posted in MH = 1 for which the model predicted 1, False positives (FP) are users with posted in MH = 0 for which the model predicted 1, and False negatives (FN) are users with posted in MH = 1 for which the model predicted 0.

### References

1. R Core Team. R: A Language and Environment for Statistical Computing. Vienna, Austria; 2022. Available: https://www.r-project.org
