# Supplementary material for: Posting patterns in peer online support forums and their associations with emotions and mood in bipolar disorder: Exploratory analysis
Source: PLoS One. 2023 Sep 25;18(9):e0291369. doi: 10.1371/journal.pone.0291369 (PMC10519601; doi:10.1371/journal.pone.0291369)
Supplement: S3 Appendix — (DOCX) [file pone.0291369.s005.docx]

S3 Appendix. Assumptions checking for the logistic regression model with controls and LIWC variables.

## Overview of logistic regression model assumption checks

Assumptions for logistic regression were checked according to Field et al. [1]. As summarised in S3 Table, most assumptions were met satisfactorily. Future work could try to address the lack of a linear relationship between some predictors in the model by applying more complex models with splines or nonlinear transformations of the predictors.

S3 Table. Logistic regression model assumption checks.

| Assumption | Check results | Assumption met? |
| --- | --- | --- |
| Independence of errors | Given the study design, all cases (the users) are independent, as only a single measurement per user is used for each variable. | ✓ |
| No multicollinearity | Some multicollinearity is present in the model, but it is well below empirical thresholds for potentially harmful collinearity (see below for details). | ✓ |
| Linear relationship between continuous predictors and the logit of the outcome variable | All predictors except for age, and active days showed a linear relationship with the logit of the outcome variable (see below for details). | (✓) |
| Enough cases for all independent variable combinations | Could not be checked via crosstabulation since all variables except gender were continuous. However, no unreasonably inflated standard errors were detected [1]. | ✓ |

## Multicollinearity

According to S4 Table, there is little to no relationship between most predictors in pairwise comparisons. Weak to medium positive correlations (0.2 < Spearman’s rho ≤ 0.5) exist between anxiety, sadness, and feminine gender; first-person pronoun use, positive emotion, anxiety, and feminine gender; and anger, masculine gender, and activity. Weak negative correlations exist between anger and first-person pronoun use and active days and activity. The variance inflation factor (VIF) of all predictors in the model is well below the empirical critical threshold of 10 and the mean VIF of 1.13 is only marginally above the empirical threshold of 1 [1]. Belsley et al.’s [2] condition number kappa *κ* of 21.79 (calculated via collin.fnc from the languageR package) is within the range of medium collinearity of 6 < *κ* < 30 and below the ≥ 30 threshold of potentially harmful collinearity suggested by Baayen [3].

S4 Table. Pairwise correlations (Spearman’s rho) of predictive variables and variance inflation factor (VIF).

|  | anxiety | sad-ness | anger | 1^st^ pers. sg. | age | gender | active days | activity | VIF |
| --- | --- | --- | --- | --- | --- | --- | --- | --- | --- |
| posemo | -0.03 | 0.09 | -0.16 | 0.25 | -0.06 | -0.17 | -0.05 | -0.11 | 1.08 |
| anxiety | 1.00 | 0.24 | 0.04 | 0.21 | -0.03 | -0.27 | -0.13 | 0.03 | 1.12 |
| sadness |  | 1.00 | 0.16 | 0.18 | 0.00 | -0.15 | -0.04 | 0.06 | 1.12 |
| anger |  |  | 1.00 | -0.22 | -0.04 | 0.24 | 0.06 | 0.27 | 1.08 |
| 1^st^ pers. sg. |  |  |  | 1.00 | -0.10 | -0.41 | -0.14 | -0.19 | 1.31 |
| age |  |  |  |  | 1.00 | 0.00 | -0.13 | 0.11 | 1.04 |
| gender |  |  |  |  |  | 1.00 | 0.15 | 0.11 | 1.27 |
| active days |  |  |  |  |  |  | 1.00 | -0.21 | 1.10 |
| activity |  |  |  |  |  |  |  | 1.00 | 1.07 |

##

## Testing for linearity of the logit

The linear relationship between continuous predictors and the outcome variable posted_in_MH was tested following Field et al. [1] by adding the natural logarithm of all continuous predictors to the model. According to Field et al. [1], a predictor is linearly related to the logarithm of the outcome if the logarithmic predictor term is not significant. As S5 Table shows, this is the case for all predictors except age and active days.

S5 Table. Results for the glm regression model including controls and LIWC variables and logarithmic terms for continuous predictors.

| **Model with controls and LIWC variables** | Est/Beta | | SE | z | | *P* | |
| --- | --- | --- | --- | --- | --- | --- | --- |
| (Intercept) | -1.49 | | 0.54 | -2.75 | | 0.01 | |
| age | -0.05 | | 0.05 | -1.00 | | 0.32 | |
| gender | -0.79 | | 0.06 | -13.93 | | <.001 | |
| active days | 0.01 | | 0.00 | 8.96 | | <.001 | |
| activity | 0.33 | | 0.04 | 8.99 | | <.001 | |
| posemo | 0.23 | | 0.10 | 2.28 | | 0.02 | |
| anxiety | 0.85 | | 0.17 | 5.12 | | <.001 | |
| anger | -0.47 | | 0.14 | -3.45 | | <.001 | |
| sadness | 0.50 | | 0.17 | 2.88 | | 0.004 | |
| 1^st^ pers. sg. | 0.60 | | 0.19 | 3.22 | | 0.001 | |
|  | | | | | | | |
| log_age | 0.00 | | 0.00 | -8.35 | | <.001 | |
| log_active_days | -0.10 | | 0.01 | -8.64 | | <.001 | |
| log_activity | -0.06 | | 0.03 | -1.79 | | 0.07 | |
| log_posemo | 0.00 | | 0.30 | 0.00 | | 1.00 | |
| log_anxiety | 0.25 | | 0.10 | 2.43 | | 0.01 | |
| log_anger | -0.02 | | 0.24 | -0.07 | | 0.95 | |
| log_sadness | -0.21 | | 0.07 | -3.05 | | 0.002 | |
| log_1^st^ pers. sg. | -1.49 | | 0.54 | -2.75 | | 0.01 | |
| Model fit | | | | | | | |
| Pseudo R^2^ | | Hosmer and Lemeshow | | | Cox and Snell | | Nagelkerke |
|  | | 0.064 | | | 0.066 | | 0.101 |
| Key: p-values for fixed effects calculated using Satterthwaites approximations. Model equation: posted_in_MH ~ age + gender + active days + activity + posemo + anxiety + anger + sadness + 1^st^ pers. sg. + log_posemo + log_anxiety + log_anger + log_sadness + log_1^st^ pers. sg. + log_ age + log_active_days + log_activity, family = binomial(link="logit") n = 10,031 subjects = total obs (126 subjects get deleted because the mean of one of their LIWC scores is zero, so the log is undefined) | | | | | | | |

### References

1. Field A, Miles J, Field Z. Discovering Statistics Using R. SAGE Publications Inc.; 2012.

2. Belsley DA, Kuh E, Welsch RE. Regression Diagnostics: Identifying Influential Data and Sources of Collinearity. Wiley Seri. New York: Wiley; 1980. doi:10.2307/3150985

3. Baayen H. Analyzing Linguistic Data: A Practical Introduction to Statistics using R. Regression Modeling. Cambridge: Cambridge University Press; 2008. doi:10.1201/9781420091984
