# Supplementary material for: Posting patterns in peer online support forums and their associations with emotions and mood in bipolar disorder: Exploratory analysis
Source: PLoS One. 2023 Sep 25;18(9):e0291369. doi: 10.1371/journal.pone.0291369 (PMC10519601; doi:10.1371/journal.pone.0291369)
Supplement: S4 Appendix — (DOCX) [file pone.0291369.s006.docx]

S4 Appendix. Probability calculation for the gender coefficient.

The 0.56 intercept coefficient represents odds of exp(0.56) = 1.75 which in turn represents an overall probability of posting in MH subreddits of p = 1.75 / (1 + 1.75) = 0.64 which is our models’ best estimate for the grand mean for the data. Using our model coefficients, the log odds of feminine users posting in MH subreddits is 0.56 + 0.79 = 1.35 and for masculine users is 0.57 – 0.79 = -0.23. The odds of posting in MH subreddits for each gender are exp(1.35) = 3.86 for feminine and
exp(-0.23) = 0.79 for masculine. The probability of posting in MH subreddits for feminine users is 3.86/(1 + 3.86) = 0.79 and for masculine users is 0.79/(1 + 0.79) = .44. Therefore the -0.79 beta coefficient represents an estimated 13% increase in the likelihood to post in MH subreddits for feminine users compared to the grand mean and a 20% decrease for masculine users. In other words, feminine users are 33% more likely to post in MH subreddits than masculine users. This change in the likelihood to post in MH subreddits is reflected in differences between the actual sample means feminine = 0.84 and masculine = 0.69 around the actual sample grand mean of 0.77.
