# Supplementary material for: Posting patterns in peer online support forums and their associations with emotions and mood in bipolar disorder: Exploratory analysis
Source: PLoS One. 2023 Sep 25;18(9):e0291369. doi: 10.1371/journal.pone.0291369 (PMC10519601; doi:10.1371/journal.pone.0291369)
Supplement: S5 Appendix — (DOCX) [file pone.0291369.s007.docx]

S5 Appendix. Regression results for subsampled dataset with gender-balanced outcome groups.

For gender balancing a random sample of users with the over-represented gender in the outcome group was selected to match the sample size of the underrepresented gender, e.g., 896 users with masculine gender identity were randomly selected from the total of 1,416 users with masculine gender identity who never posted in MH subreddits (see S6 Table). Also, for the gender-balanced dataset, the logistic regression model including the LIWC variables had a better fit than the model with only the control predictors (S7 Table). As S8 Table shows, the results for the gender-balanced sample did not change substantially compared to the results of the original sample in Table 4. Specifically, the effect of positive emotion stayed almost exactly the same (95% CI [0.03 - 0.08]). The effects of sadness and anxiety decreased in significance level from *P* < .001 to *P* = .002 and slightly in effect size: The 95% CI of the anxiety coefficient decreased to [0.20 – 0.83] from previously
[0.32 – 0.91] and sadness to [0.13 – 0.62] from previously [0.18 – 0.63].

S6 Table. Sample sizes according to outcome and gender before and after balancing for gender.

| posted_in_MH | Gender | Before gender balancing | After gender balancing |
| --- | --- | --- | --- |
| posted_in_MH = 0 | f | 896 | 896 |
|  | m | 1,416 | 896 |
| posted_in_MH = 1 | f | 4,696 | 3,150 |
|  | m | 3,150 | 3,150 |
| Total sample size (#users) |  | 10,309 | 8,092 |
| % of users with posted_in_MH = 1 |  | 77.2 | 77.9 |

S7 Table. Model fit comparison for controls only and controls with LIWC variables for gender-balanced dataset.

| **Model name** | **Nested/ simpler Model** | **Predictors added** | **Model fit** | | | | **LRT against nested** | |
| --- | --- | --- | --- | --- | --- | --- | --- | --- |
|  |  |  | **AIC** | **BIC** | **LL** | **df** | **df** | **X2** |
| Controls | - | age, gender, active days, activity | 8465 | 8500 | -4227 | 8087 |  |  |
| Controls + LIWC | Controls | posemo, anxiety, anger, sadness, 1^st^ pers. sg. | 8418 | 8488 | -4119 | 8082 | 5 | 56.34, *P* <.001 |

S8 Table. Results for the glm regression model including controls and LIWC variables for gender-balanced dataset.

| **Model  Controls + LIWC** | | Est/Beta | SE | 95% CI | | z | *P* | Odds ratio (exp(coeff)) | |
| --- | --- | --- | --- | --- | --- | --- | --- | --- | --- |
|  |  |  |  |  |  |  |  | Est/Beta | 95% CI |
| (Intercept) | | 0.15 | 0.19 | -0.23 – 0.53 | | 0.78 | 0.43 | 1.16 | 0.80 – 1.70 |
| age | | -0.0005 | 0.003 | -0.01 – 0.01 | | -0.18 | 0.86 | 1.00 | 0.99 – 1.01 |
| gender | | 0.06 | 0.06 | -0.06 –0.18 | | 0.91 | 0.36 | 1.06 | 0.94 – 1.19 |
| active days | | 0.0003 | 0.00003 | 0.0003 – 0.0004 | | 10.16 | <.001 | 1.00 | 1.00 – 1.00 |
| activity | | -0.01 | 0.01 | -0.02 – 0.01 | | -1.18 | 0.24 | 0.99 | 0.98 – 1.01 |
| posemo | | 0.06 | 0.01 | 0.04 – 0.08 | | 4.98 | <.001 | 1.06 | 1.04 – 1.09 |
| anxiety | | 0.51 | 0.16 | 0.20 – 0.83 | | 3.17 | 0.002 | 1.67 | 1.23 – 2.31 |
| anger | | -0.07 | 0.05 | -0.16 – 0.02 | | -1.55 | 0.12 | 0.93 | 0.85 – 1.02 |
| sadness | | 0.38 | 0.12 | 0.13 – 0.62 | | 3.04 | 0.002 | 1.46 | 1.15 – 1.87 |
| 1^st^ pers. sg. | | 0.02 | 0.02 | -0.02 – 0.05 | | 0.84 | 0.40 | 1.02 | 0.98 – 1.05 |
| Model fit | | | | | | | | | |
| Pseudo R^2^ | Hosmer and Lemeshow | | | | Cox and Snell | | | | Nagelkerke |
|  | 0.019 | | | | 0.019 | | | | 0.030 |
| Key: p-values for fixed effects calculated using Satterthwaites approximations. Confidence Intervals have been calculated using the Wald method. Model equation: glm(posted_in_MH ~ age + gender + active days + activity + posemo + anxiety + anger + sadness + 1^st^ pers. sg., family = binomial(link="logit")) | | | | | | | | | |
