# Supplementary material for: Posting patterns in peer online support forums and their associations with emotions and mood in bipolar disorder: Exploratory analysis
Source: PLoS One. 2023 Sep 25;18(9):e0291369. doi: 10.1371/journal.pone.0291369 (PMC10519601; doi:10.1371/journal.pone.0291369)
Supplement: Attachment — Submitted filename: BipolarPostingMood_PLOS_R1_ReviewerResponse.pdf [file pone.0291369.s008.pdf]

PONE-D-22-26677
Posting patterns in peer online support forums and their associations with emotions and mood in bipolar disorder: Exploratory analysis
PLOS ONE

Response to Reviewers

For ease of checking the revisions, we have added the reviewer and comment number to all changes in the Track Changes version of the revised manuscript and copied them in this response letter.

Reviewer 1 (Grigori Sidorov)'s comments to the authors

Contribution:
• User identification and dataset construction in Reddit posts
• Study of relationships between posting patterns and people's emotions
Our response: Thank you for recognising the contributions of our paper.

Positive:
• A complete introduction is given
• Research questions are presented and addressed very well
• Paper is well written and easy to follow
• Sufficient details are given and claims are justified with proper citations
• The results and analysis are tabulated and discussed in detail
Our response: Thank you very much!

Negative:
1. The body text alignment need correction
Our response:
We have reformatted the manuscript according to PLOS ONE's style requirements.

2. Use [] for citations instead of ()
Our response:
We now use [] instead of () according to the PLOS ONE citation style.

3. Figure 1 is not referred in text body
Our response:
We have fixed the references to Figure 1.
Our manuscript revision:
Fig. 1 shows that two thirds of the 19,685 identified Reddit users who disclosed a BD diagnosis posted in both MH and non-MH subreddits.

4. What are the main contributions of your work?
Our response:
We have added a list of contributions at the end of the introduction.
Our manuscript revision:

Aim, research questions, and contributions

[…]

This study makes the following contributions to MH research:

- Provides the first quantitative evidence on the posting behaviour of people with a BD diagnosis and how demographic and emotional factors may impact posting in MH forums specifically (see Fig. 2 for a visual summary of the results)
- Demonstrates differences in the emotional content of MH vs non-MH subreddit posts
- Shows how to use established NLP methods to provide quantitative data and generate findings relevant for MH researchers and professionals
- Provides a large dataset of 21M Reddit posts by 20K Reddit users with a self-reported BD diagnosis that is available for future research

5. The use of LR model is not presented well, how did you model the task?

Our response:
Thank you for letting us know that this was not clear. We elaborated on the LR model in the added S2 Appendix referred to in the methods section. We now also refer in the results section to the assumption checks we conducted for the logistic regression model in S3 Appendix.
Our manuscript revisions:
To address RQ3, a logistic regression model was fitted via the R [71, Windows version 4.1.0] glm function to predict whether a user had posted in MH subreddits based on the mean LIWC variable scores of their non-MH subreddit posts and some control variables. See S2 Appendix for method details.

[…]

S2 Table provides descriptive statistics of the dependent variables. Logistic regression assumptions were checked according to Field et al. [86] (see S3 Appendix for details).

S2 Appendix. Method details for the logistic regression model with controls and LIWC variables.

Rationale for model selection

A logistic regression model was most appropriate to address RQ3 because it reveals which predictors significantly impact on the outcome and their effect sizes. While more complex models may yield higher accuracy, they are more difficult to interpret and therefore not suitable to address RQ3.

Model estimation

The logistic regression model was fitted via the R (R Core Team, 2022, Windows version 4.1.0) glm function with the following specification:

model.liwc_w_controls <- glm(posted in MH ~ age + gender + active_days + activity + posemo + anxiety + anger + sadness + first_pers_sg, data = df, family = binomial(link="logit"))

This corresponds to describing the probability of a user having posted in MH subreddits via the following equation in vector notation:

$$p(\text{posted in MH}) = \frac{1}{1 + e^{-(\beta_0+\beta_1\text{age}+\beta_2\text{gender}+\beta_3\text{active\_days}+\beta_4\text{activity}+\beta_5\text{posemo}+\beta_6\text{anxiety}+\beta_7\text{anger}+\beta_8\text{sadness}+\beta_9\text{first\_pers\_sg})}}$$

The model parameters β_0 (intercept) and predictor coefficients $\beta_1 - \beta_9$ are fitted via maximum likelihood estimation.

The model was estimated using all 10,158 users which met the eligibility criteria and for which age and gender information was available. The outcome variable *posted in MH* was imbalanced with

7,846 (77.2%) of users having posted in MH subreddits (posted in MH = 1) and 2,312 (22.8%) of users only having posted in non-MH subreddits (posted in MH = 0). As S1 Fig. shows, the fitted model outperforms the majority class baseline that always predicts posted in MH = 1 for every user in the dataset for all recall levels except 1.0. Balancing the outcome variable by randomly subsampling 2,312 users with posted in MH = 1 only changed the intercept but not the predictor coefficients substantially, as expected.

The model was used to estimate the impact of each predictor on the outcome for the known users.

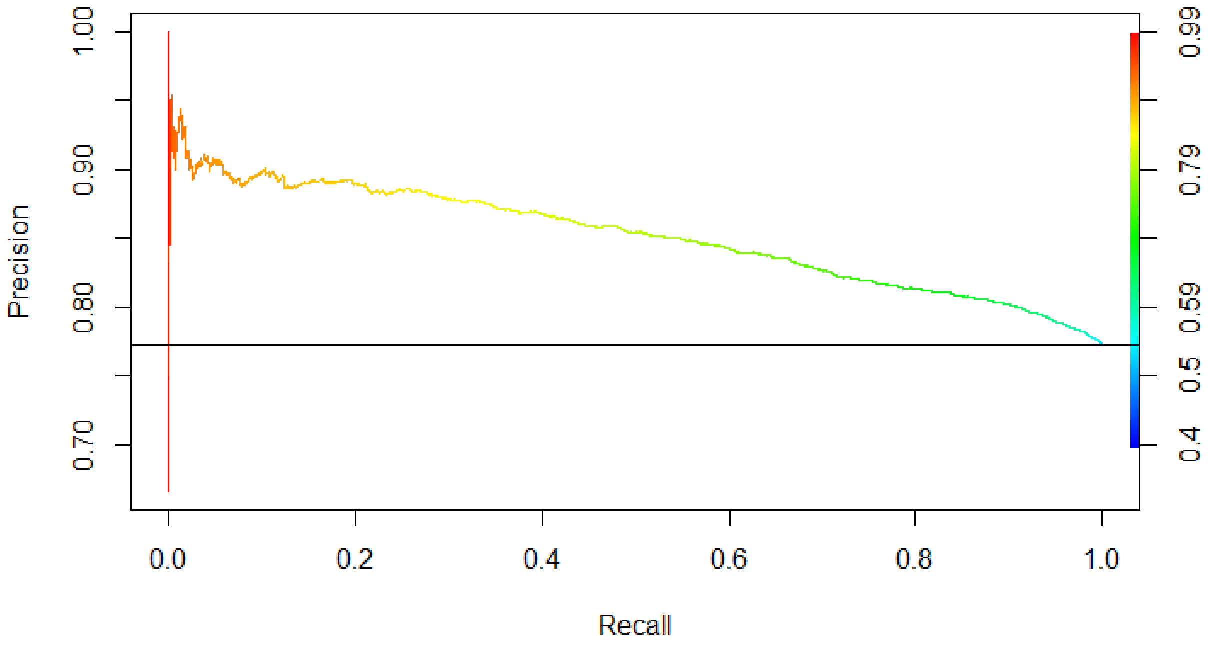

S1 Fig. Precision-recall plot for the glm regression model with controls and LIWC variables applied to the 10,158 users it was estimated on. Precision = TP / (TP + FP), Recall = TP (TP + FN), where True positives (TP) are users with posted in MH = 1 for which the model predicted 1, False positives (FP) are users with posted in MH = 0 for which the model predicted 1, and False negatives (FN) are users with posted in MH = 1 for which the model predicted 0.

References

Andy, A., Sherman, G. and Guntuku, S. C. (2022) 'Understanding the expression of loneliness on Twitter across age groups and genders', *PLoS ONE*, 17(9 September), pp. 1–19. doi: 10.1371/journal.pone.0273636.

Boyd, R. L. and Pennebaker, J. W. (2017) 'Language-based personality: a new approach to personality in a digital world', *Current Opinion in Behavioral Sciences*, 18, pp. 63–68. doi: 10.1016/j.cobeha.2017.07.017.

Canales, L. and Martínez-barco, P. (2014) 'Emotion Detection from text: A Survey', in *Proceedings of the Workshop on Natural Language Processing in the 5th Information Systems Research Working Days*. Association for Computational Linguistics, pp. 37–43.

De Choudhury, M. *et al.* (2017) 'Gender and Cross-Cultural Differences in Social Media Disclosures of Mental Illness', in *Proceedings of the 2017 ACM Conference on Computer Supported Cooperative Work and Social Computing*, pp. 353–369. doi: 10.1145/2998181.2998220.

Cohan, A. *et al.* (2018) 'SMHD: A Large-Scale Resource for Exploring Online Language Usage for Multiple Mental Health Conditions', in *Proceedings of the 27th International Conference on Computational Linguistics (COLING)*, pp. 1485–1497. Available at:

https://www.aclweb.org/anthology/C18-1126.

Coppersmith, G. *et al.* (2015) 'From ADHD to SAD: Analyzing the Language of Mental Health on Twitter through Self-Reported Diagnoses', pp. 1–10. doi: 10.3115/v1/w15-1201.

Coppersmith, G., Dredze, M. and Harman, C. (2014) 'Quantifying Mental Health Signals in Twitter', in *Proceedings of the Workshop on Computational Linguistics and Clinical Psychology: From Linguistic Signal to Clinical Reality*, pp. 51–60. doi: 10.3115/v1/W14-3207.

Demszky, D. *et al.* (2020) 'GoEmotions : A Dataset of Fine-Grained Emotions', in *Proceedings of the 58th Annual Meeting of the Association for Computational Linguistics*, pp. 4040–4054.

Dzogang, F., Lansdall-Welfare, T. and Cristianini, N. (2016) 'Seasonal Fluctuations in Collective Mood Revealed by Wikipedia Searches and Twitter Posts', *IEEE International Conference on Data Mining Workshops, ICDMW*, 0, pp. 931–937. doi: 10.1109/ICDMW.2016.0136.

Dzogang, F., Lightman, S. and Cristianini, N. (2018) 'Diurnal variations of psychometric indicators in twitter content', *PLoS ONE*, 13(6). doi: 10.1371/journal.pone.0197002.

Ekkekakis, P. (2013) *The Measurement of Affect, Mood, and Emotion*, *Panteleimon Ekkekakis*. Cambridge University Press. doi: 10.1176/pn.39.2.0031b.

Ekman (1992) 'An argument for basic emotions', *Cognition and Emotion*, 6, pp. 169–200.

Entwistle, C., Marceau, E. and Boyd, R. L. (2022) 'Personality disorder and verbal behavior', *Handbook of language analysis in psychology.*, pp. 335–356. Available at: http://ovidsp.ovid.com/ovidweb.cgi?T=JS&PAGE=reference&D=psyc18&NEWS=N&AN=2022-08338-017.

Fineberg, S. K. *et al.* (2016) 'Self-reference in psychosis and depression: A language marker of illness', *Psychological Medicine*, 46(12), pp. 2605–2615. doi: 10.1017/S0033291716001215.

Furnham, A. (1983) 'Social psychology as common sense', *Bulletin of the British Psychological Society*, 36, pp. 105–109.

Golder, S. A. and Macy, M. W. (2011) 'Diurnal and seasonal mood vary with work, sleep, and daylength across diverse cultures', *Science*, 333(6051), pp. 1878–1881. doi: 10.1126/science.1202775.

Harvey, D. *et al.* (2022) 'Natural Language Processing Methods and Bipolar Disorder: Scoping Review', *JMIR Mental Health*, 9(4). doi: 10.2196/35928.

Ireland, M. and Iserman, M. (2018) 'Within and Between-Person Differences in Language Used Across Anxiety Support and Neutral Reddit Communities', in *Proceedings of the Fifth Workshop on Computational Linguistics and Clinical Psychology: From Keyboard to Clinic,* pp. 182–193. doi: 10.18653/v1/w18-0620.

Kahn, J. H. *et al.* (2007) 'Measuring emotional expression with the Linguistic Inquiry and Word Count', *American Journal of Psychology*, 120(2), pp. 263–286. doi: 10.2307/20445398.

Kennedy, B. *et al.* (2022) 'Text Analysis for Psychology: Methods, Principles, and Practices', *Guilford Press*, pp. 3–33.

Köper, M., Kim, E. and Klinger, R. (2017) 'IMS at EmoInt-2017: Emotion Intensity Prediction with Affective Norms, Automatically Extended Resources and Deep Learning', in *Proceedings of the 8th Workshop on Computational Approaches to Subjectivity, Sentiment and Social Media Analysis (WASSA)*, pp. 50–57. doi: 10.18653/v1/W17-5206.

Newell, E. E. *et al.* (2018) 'You Sound So Down: Capturing Depressed Affect Through Depressed Language', *Journal of Language and Social Psychology*, 37(4), pp. 451–474. doi:

10.1177/0261927X17731123.

O’Dea, B. *et al.* (2021) ‘The relationship between linguistic expression in blog content and symptoms of depression, anxiety, and suicidal thoughts: A longitudinal study’, *PLoS ONE*, 16(5 May), pp. 1–17. doi: 10.1371/journal.pone.0251787.

Park, A. and Conway, M. (2017) ‘Longitudinal changes in psychological states in online health community members: Understanding the long-term effects of participating in an online depression community’, *Journal of Medical Internet Research*, 19(3). doi: 10.2196/jmir.6826.

Pennebaker, J. W. *et al.* (2015) *The Development and Psychometric Properties of LIWC2015*. Austin. doi: 10.15781/T29G6Z.

Pennebaker, J. W. (2022) ‘Computer-Based Language Analysis as a Paradigm Shift’, *Handbook of language analysis in psychology*, pp. 576–587.

Pennebaker, J. W. and Beall, S. K. (1986) ‘Confronting a Traumatic Event. Toward an Understanding of Inhibition and Disease’, *Journal of Abnormal Psychology*, 95(3), pp. 274–281. doi: 10.1037/0021-843X.95.3.274.

Pérez, J. M., Giudici, J. C. and Luque, F. (2021) ‘pysentimiento: A Python Toolkit for Sentiment Analysis and SocialNLP tasks’, pp. 1–4. Available at: http://arxiv.org/abs/2106.09462.

R Core Team (2022) *R: A Language and Environment for Statistical Computing*. Vienna, Austria. Available at: https://www.r-project.org.

Sekulić, I. *et al.* (2018) ‘Not Just Depressed: Bipolar Disorder Prediction on Reddit’, in *9th Workshop on Computational Approaches to Subjectivity, Sentiment and Social Media Analysis (WASSA)*. Brussels, pp. 72–78. doi: 10.18653/v1/P17.

Sekulic, I. and Strube, M. (2019) ‘Adapting Deep Learning Methods for Mental Health Prediction on Social Media’, pp. 322–327. doi: 10.18653/v1/d19-5542.

Stone, J. A. and Ryerson, N. C. (2022) ‘Tweeting about alcohol: Exploring differences in Twitter sentiment during the onset of the COVID-19 pandemic’, *Plos One*, 17(11), p. e0276863. doi: 10.1371/journal.pone.0276863.

Tackman, A. M. *et al.* (2018) ‘Depression, Negative Emotionality, and Self-Referential Language: A Multi-Lab, Multi-Measure, and Multi-Language-Task Research Synthesis’, *Journal of Personality and Social Psychology*, (March). doi: 10.1037/pspp0000187.

Tausczik, Y. R. and Pennebaker, J. W. (2010) ‘The psychological meaning of words: LIWC and computerized text analysis methods’, *Journal of Language and Social Psychology*, 29(1), pp. 24–54. doi: 10.1177/0261927X09351676.

Tay, D. and Qiu, H. (2022) ‘Modeling Linguistic (A)Synchrony: A Case Study of Therapist–Client Interaction’, *Frontiers in Psychology*, 13(May). doi: 10.3389/fpsyg.2022.903227.

Improvements:

6. The use of LR model to address RQ3 could be elaborated more. From a NLP perspective it requires to describe how it was trained, what statistics of dataset was used and more detail about the procedure.

Our response:
We have added the requested details on the LR model in the new S2 Appendix. See our response to your Comment 5.

7. The contributions of the article could be listed

Our response:
Thank you for this great suggestion. We now list our contributions at the end of the introduction. See our response to your Comment 4.

8. More graphical charts and figure would help for a better representation of your work

Our response:
Thank you for this suggestion! We have added a visual summary of our results in Fig 2.

Manuscript revisions:

Results summary

Fig. 2 provides a visual summary of all results. Two thirds of the identified 19,685 users with a self-reported BD diagnosis posted in both MH and non-MH subreddits, while 24% never posted in MH subreddits. Users who posted in both MH and non-MH subreddits used more first-person singular pronouns and exhibited more sadness and anxiety, but less positive emotion in their MH subreddit posts. Feminine gender and expressing more anxiety, sadness, and positive emotion in non-MH subreddit posts were significant predictors for posting in MH subreddits.

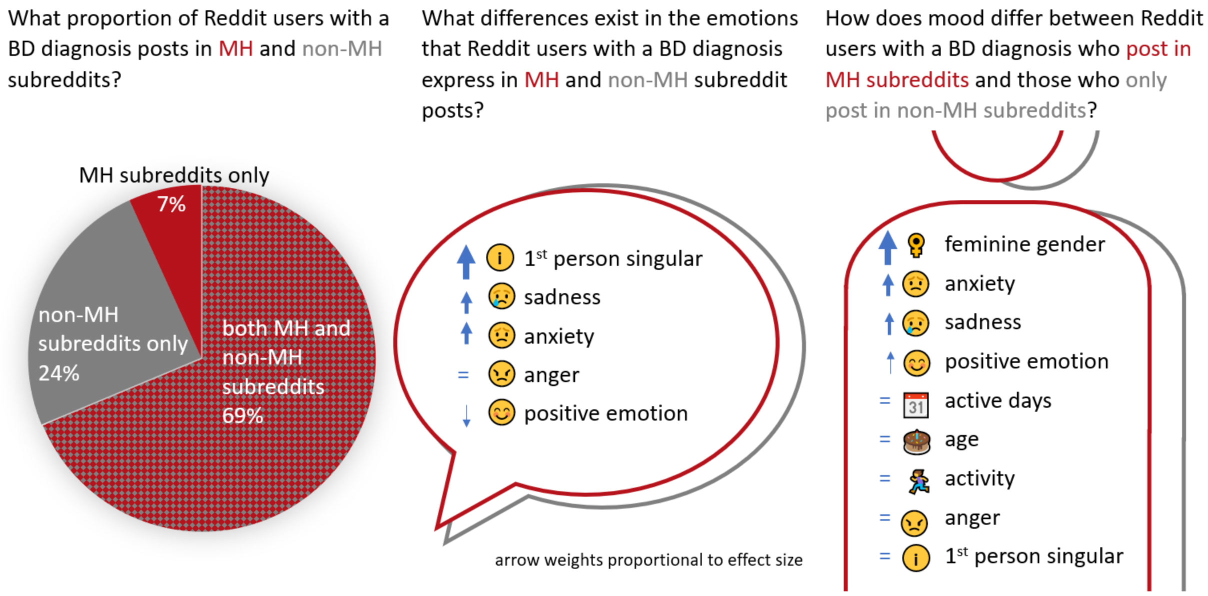

Fig. 2. Visual summary of the results.

9. Since you have explored emotion, you may consider exploring Symanto API as well for future work, where it will provides you with tool for extract if the text is rational or emotional. Sentiments Analysis, Emotion detection, and psychological tools are other interesting and related tools for you.

Our response:
Thank you for suggesting Symanto API. We have had a look at it and it looks certainly interesting. From their subscription models, we understand that students can use the APIs for free for three months, which would include up to 100,000 requests/month for Premium APIs, which includes the Ekman emotion analysis. Unfortunately, this would not suffice to analyse the 6.5M posts used in this paper and there is no more funding available in the PhD project to pay for additional requests. We will consider using the Symanto API in future research projects where further funding is available.

Reviewer 2 (anonymous) comments to the authors

1. The study makes no contribution to the field of natural language processing.

Our response:

We agree that our study makes no contributions to NLP, which we did not intend to claim. The contributions, which we now list at the end of the introduction (see Reviewer 1 Comment 4), lie in the application of established NLP methods to generate findings that are novel in the field of MH research.

2. For emotion text analysis, the dictionary-based LIWC method does not provide state-of-the-art NLP results. Using more relevant tools may provide different outcomes.

Our response:

We agree that using different NLP methods based on machine learning and deep learning may provide different outcomes, as we already stated in the future work section. Nevertheless, we believe that LIWC is the best choice to achieve our goal of generating the first quantitative knowledge on the relationship of emotions and posting patterns for people with a bipolar disorder diagnosis for four reasons:

(1) This type of language analysis via expert-created dictionaries is rooted in over 100 years of empirical research in the behavioural sciences and there is a large evidence base for the psychometric validity of this approach.
(2) LIWC emerged from James Pennebaker's work on narrative therapy, which is widely recognised amongst clinicians, while most of them are not familiar with machine learning and deep learning approaches.
(3) The dictionary-based LIWC approach is simple to explain and the results are transparent. It is straightforward to trace which words contribute to the emotion scores. Machine learning and deep learning methods are more complex to explain, and the results are often hard to interpret.
(4) Many social science and psycholinguistic studies used (only) LIWC to test and develop theories as we do. This provides further validation for the choice of our method and enabled a more straightforward comparison to previous work, which mainly used LIWC, too, in the discussion of RQ 2 and RQ 3.

Finally, as we already suggested in the future work section, using different NLP method to assess emotions and compare the findings with our results would be interesting. However, this may detract from the accessibility, and interest of the paper to a broader mental health audience. Therefore, we believe that this needs to be done in a separate publication targeted at an NLP audience. The aim of our paper is to provide quantitative evidence targeted at MH researchers and professionals and discuss the implications for mental health research and care, not to compare NLP methods for emotion analysis.

Our manuscript revisions:

Language analysis

LIWC (Pennebaker *et al.*, 2015) was used to obtain text-analytic measures of peoples' emotions and mood. In this study, users' emotions – usually short-lived (seconds (Ekman, 1992)), high in intensity and in response to the evaluation of a stimulus (Ekkekakis, 2013, p. 41) – are operationalised via the LIWC scores of single posts. Averaged LIWC scores of multiple posts over a longer timespan are assumed to represent users' mood, which is of longer duration, lower intensity and not in direct temporal relationship to a stimulus (Ekkekakis, 2013, pp. 44–45).

Recent NLP research focuses on emotion text analysis systems based on machine learning or deep learning methods (e.g., Köper, Kim and Klinger, 2017; Demszky *et al.*, 2020; Pérez, Giudici and Luque, 2021). Yet, LIWC was considered the most suitable emotion analysis tool for this study for four main reasons. First, this type of language analysis via expert-created dictionaries is rooted in over 100 years of empirical research in the behavioural sciences and there is a large evidence base for the

psychometric validity of this approach (Tausczik and Pennebaker, 2010; Boyd and Pennebaker, 2017; Entwistle, Marceau and Boyd, 2022; e.g., Kennedy *et al.*, 2022; Pennebaker, 2022). Particularly for the LIWC emotion categories, Kahn et al. (2007) demonstrated that the scores for positive emotion, negative emotion and sadness significantly differ in the expected ways in peoples' responses to discrete stimuli. The participants had higher positive emotion and lower negative emotion and sadness scores when writing about an amusing versus sad life experience and describing their feelings after exposure to a funny versus sad video clip. Newell et al. (2018) showed that more negative emotion, less positive emotion, and a higher use of first-person singular pronouns correlates with peoples' subjectively reported stress level and their blood pressure. Moreover, LIWC emotion variables also correlate with established daily and seasonal mood fluctuations within individuals and for entire populations (Golder and Macy, 2011; Dzogang, Lansdall-Welfare and Cristianini, 2016; Dzogang, Lightman and Cristianini, 2018). In contrast to this, machine learning-based NLP methods require labelled training data and usually only perform well on data that is similar to the training data. While Google recently released an emotion-labelled dataset of Reddit posts (Demszky *et al.*, 2020), it is not specific to people with MH issues and therefore not suitable to train or fine-tune an emotion classifier for the aims of this study. Second, LIWC emerged from Pennebaker's work on narrative therapy (e.g., Pennebaker and Beall, 1986), which is widely recognised amongst clinicians, while most of them are not familiar with machine learning and deep learning approaches. Applied psycholinguistic research using LIWC is very active as evidenced by many studies published just in the past two years (O'Dea *et al.*, 2021; Andy, Sherman and Guntuku, 2022; Stone and Ryerson, 2022; e.g., Tay and Qiu, 2022). Third, LIWC yields results that are easy to interpret because they directly relate to the frequencies of words in the dictionaries. This is not the case for machine learning or deep learning systems whose predictions are often opaque and hard to trace back to the input (Canales and Martínez-barco, 2014). Fourth, several previous studies have applied LIWC to online posts of people with MH issues, including BD (Coppersmith, Dredze and Harman, 2014; Coppersmith *et al.*, 2015; Fineberg *et al.*, 2016; De Choudhury *et al.*, 2017; Park and Conway, 2017; Cohan *et al.*, 2018; Ireland and Iserman, 2018; Sekulić *et al.*, 2018), to the extent that most research on BD using NLP methods has used LIWC (Harvey *et al.*, 2022). This allows for a straightforward comparison of the present results with previous work in the discussion or RQ2 and RQ 3.

RQ 2: What differences exist in the emotions that Reddit users with a BD diagnosis express in MH and non-MH subreddit posts?

The comparison between users' posts in MH and non-MH subreddit posts showed that, in MH subreddits, users expressed somewhat fewer positive emotions, more anxiety and sadness, and used much more first-person singular pronouns. These findings aligned with the expectations of people with lived experience that people may mainly post in MH support forums about MH struggles and when experiencing mental distress. They also aligned with the finding from Ireland and Iserman (2018) that people used more anxious language in anxiety-focused subreddits compared to their posts in other subreddits. Increased use of first person singular pronouns has been mainly linked to mental distress (Tackman *et al.*, 2018), although positive I-talk may also occur (Tackman *et al.*, 2018, pp. 35–36).

3. I am not an expert in mental health, but the research result appears to be quite straightforward.

Our response:

As we detail in the introduction and motivation for our study, there has to date been no quantitative study that investigated the relationship of posting in MH support forums and emotions for people with severe MH issues. Moreover, there is a lack of evidence in general on which people with a BD diagnosis engage in MH support forums. What seems 'common sense' is actually only so when it has

been empirically demonstrated (Furnham, 1983). Therefore, although our results may be plausible given the expectations from people with lived experience of BD and related studies, they are still very valuable and important for MH researchers and professionals as they provide robust quantitative evidence for these questions for the first time. We think that our paper fits very well with PLOS ONE's journal standards that focus on scientifically rigorous research, regardless of novelty.

Our manuscript revision:

Implications for research

In general, a better understanding of people with a BD diagnosis who post on Reddit is important to contextualise other research on this kind of data, with Reddit being one of the most frequently used data sources for research on BD using NLP methods (Harvey *et al.*, 2022). More specifically, the findings for RQ 2 demonstrated substantial differences in LIWC scores between posts in MH and non-MH subreddits. Although expected, this had not been empirically tested so far. Providing quantitative evidence for this is important because what seems 'common sense' is actually only so when it has been empirically demonstrated (Furnham, 1983). The RQ2 findings provide strong support for all studies relying on emotion text analysis, specifically comparative language analyses and detection systems of people with and without MH issues, to only consider posts in a comparable context as done in more recent research (Cohan *et al.*, 2018; Sekulić *et al.*, 2018; Sekulic and Strube, 2019), compared to earlier work (Coppersmith, Dredze and Harman, 2014; Coppersmith *et al.*, 2015).

4. The author covers and refers to prior work in a methodical manner, and the statistical analysis appears to be valid.

Our response: Thank you very much!

5. I believe that the author should have compared user behavior over several time intervals. In particular, How was the user's depression represented in MH and non-MH postings on a particular day or week when they experienced it?

Our response:
Thank you for this suggestion. We agree that it would be interesting to correlate users' depressive or (hypo-)manic symptoms with the emotions expressed in their online posts. However, this would require users' self-reported or clinician-assessed depression and (hypo-)mania scores, which are not available for the Reddit users included in our dataset.

We already suggested in the paper that future work should consider this by looking at what effects engaging with online MH support forums has on users' mood as well as on person-centred holistic wellbeing. Following your suggestion, we clarified that a longitudinal design would be most useful for this.

Our manuscript revision:
Future research could extend the present findings by looking at what effects engaging with online MH support forums has on users' mood as well as on person-centred holistic wellbeing in longitudinal studies.

Minors:

6. Line: 264, 267: Error! Reference source not found

Our response:
Thank you for pointing this out. We have fixed the references to Figure 1 (see also Reviewer 1, Comment 3).

Our manuscript revision:
Fig. 1 shows that two thirds of the 19,685 identified Reddit users who disclosed a BD diagnosis posted in both MH and non-MH subreddits. One quarter never posted in MH subreddits and only 6.8% posted exclusively in MH subreddits. The majority (54.7%) of users in the S-BiDD dataset were active contributors in both MH and non-MH subreddits.

7. Line 291: formatting issue

Our response:
We have fixed the formatting issue.

Editor's comments to the authors

1. Your study deal with a very important subject.
2. Your paper contains many well-written components (e.g., the introduction chapter, research questions, results, and analysis).

Our response: Thank you very much!

However, both reviewers recommend major revisions.
3. They recommend using additional and more suitable tools for your research.
Our response:
Reviewer 1 suggested a commercial NLP tool for us to use in future work but not necessarily the present paper (see Reviewer 1, Comment 9). Reviewer 2 suggested to use more state-of-the-art NLP tools in the present paper. As we argue in more detail in response to Comment 2 by Reviewer 2 above, we believe that LIWC is the most suitable tool for our research to generate novel findings for mental health research. While comparing our results with machine learning-based NLP methods would be interesting, it may detract from the accessibility of the paper for a broader general MH audience, which the paper targets. Therefore, such a comparison of methods would be more suited to a separate publication targeted at an NLP audience.

Additionally, we would like to point out that PLOS ONE has published many studies that only used LIWC to measure emotions, including these three publications within the last two years:

74. O'Dea B, Boonstra TW, Larsen ME, Nguyen T, Venkatesh S, Christensen H. The relationship between linguistic expression in blog content and symptoms of depression, anxiety, and suicidal thoughts: A longitudinal study. PLoS One. 2021;16: 1–17. doi:10.1371/journal.pone.0251787
75. Stone JA, Ryerson NC. Tweeting about alcohol: Exploring differences in Twitter sentiment during the onset of the COVID-19 pandemic. PLoS One. 2022;17: e0276863. doi:10.1371/journal.pone.0276863
76. Andy A, Sherman G, Guntuku SC. Understanding the expression of loneliness on Twitter across age groups and genders. PLoS One. 2022;17: 1–19. doi:10.1371/journal.pone.0273636

4. They wrote that various points in your paper are not clear and not elaborated enough (many details were given by Reviewer 1).

Our response:
We have clarified the points raised by the reviewers as indicated in our responses to their comments.

5. According to Reviewer 2, your study does not provide either NLP contributions or state-of-the-art results.

Our response:
Our paper does not claim or aim to make NLP contributions or provide state-of-the-art results on emotion analysis. Instead, our contributions are to provide novel quantitative knowledge in the field of mental health research by applying established NLP methods to understand more about an important but under-researched area. We added a list of contributions to the paper as suggested by Reviewer 1 in Comment 7 to clarify this. See also our response to Comment 1 of Reviewer 2.

6. Both reviewers also report various types of minor corrections.
Our response:
We fixed all minor corrections pointed out by the reviewers.
We hope that you are satisfied with our substantive revisions of the manuscript. We would like to thank you and the reviewers again for the valuable feedback, which has helped us to improve the paper.

Journal requirements

1. Please ensure that your manuscript meets PLOS ONE's style requirements, including those for file naming. The PLOS ONE style templates can be found at https://journals.plos.org/plosone/s/file?id=wjVg/PLOSOne_formatting_sample_main_body.pdf and https://journals.plos.org/plosone/s/file?id=ba62/PLOSOne_formatting_sample_title_authors_affiliations.pdf

Our response:
We have formatted our manuscript according to PLOS ONE's style requirements.

2. We note that you have indicated that data from this study are available upon request. PLOS only allows data to be available upon request if there are legal or ethical restrictions on sharing data publicly. For more information on unacceptable data access restrictions, please see http://journals.plos.org/plosone/s/data-availability#loc-unacceptable-data-access-restrictions.

In your revised cover letter, please address the following prompts:

a) If there are ethical or legal restrictions on sharing a de-identified data set, please explain them in detail (e.g., data contain potentially sensitive information, data are owned by a third-party organization, etc.) and who has imposed them (e.g., an ethics committee). Please also provide contact information for a data access committee, ethics committee, or other institutional body to which data requests may be sent.

b) If there are no restrictions, please upload the minimal anonymized data set necessary to replicate your study findings as either Supporting Information files or to a stable, public repository and provide us with the relevant URLs, DOIs, or accession numbers. For a list of acceptable repositories, please see http://journals.plos.org/plosone/s/data-availability#loc-recommended-repositories.

We will update your Data Availability statement on your behalf to reflect the information you provide.

Our response (to a):
Data is only available for non-commercial research upon request after signing a data usage agreement due to its sensitive nature pertaining to severe mental health issues. Mental health issues are still highly stigmatised. The dataset could be used to build algorithms that predict whether someone likely meets criteria for bipolar disorder based on their Reddit posts. Such algorithms could cause harm to online users identified as likely suffering from a mental health issue if the predictions are made public. Moreover, companies (e.g. insurances or financial institutions) could use such algorithms to the disadvantage of applicants identified as likely suffering from mental health issues. The Lancaster University Faculty of Health and Medicine Research Ethics Committee imposed these restrictions. Researchers can request data access via the Lancaster University PURE repository at http://doi.org/10.17635/lancaster/researchdata/PLACEHOLDER (contact via rdm@lancaster.ac.uk).

3. Your ethics statement should only appear in the Methods section of your manuscript. If your ethics statement is written in any section besides the Methods, please move it to the Methods section and delete it from any other section. Please ensure that your ethics statement is included in your manuscript, as the ethics statement entered into the online submission form will not be published alongside your manuscript

Our response:
Our ethics statement was already placed in the Methods section of the manuscript.

References

Andy, A., Sherman, G. and Guntuku, S. C. (2022) 'Understanding the expression of loneliness on Twitter across age groups and genders', *PLoS ONE*, 17(9 September), pp. 1–19. doi: 10.1371/journal.pone.0273636.

Boyd, R. L. and Pennebaker, J. W. (2017) 'Language-based personality: a new approach to personality in a digital world', *Current Opinion in Behavioral Sciences*, 18, pp. 63–68. doi: 10.1016/j.cobeha.2017.07.017.

Canales, L. and Martínez-barco, P. (2014) 'Emotion Detection from text: A Survey', in *Proceedings of the Workshop on Natural Language Processing in the 5th Information Systems Research Working Days*. Association for Computational Linguistics, pp. 37–43.

De Choudhury, M. *et al.* (2017) 'Gender and Cross-Cultural Differences in Social Media Disclosures of Mental Illness', in *Proceedings of the 2017 ACM Conference on Computer Supported Cooperative Work and Social Computing*, pp. 353–369. doi: 10.1145/2998181.2998220.

Cohan, A. *et al.* (2018) 'SMHD: A Large-Scale Resource for Exploring Online Language Usage for Multiple Mental Health Conditions', in *Proceedings of the 27th International Conference on Computational Linguistics (COLING)*, pp. 1485–1497. Available at: https://www.aclweb.org/anthology/C18-1126.

Coppersmith, G. *et al.* (2015) 'From ADHD to SAD: Analyzing the Language of Mental Health on Twitter through Self-Reported Diagnoses', pp. 1–10. doi: 10.3115/v1/w15-1201.

Coppersmith, G., Dredze, M. and Harman, C. (2014) 'Quantifying Mental Health Signals in Twitter', in *Proceedings of the Workshop on Computational Linguistics and Clinical Psychology: From Linguistic Signal to Clinical Reality*, pp. 51–60. doi: 10.3115/v1/W14-3207.

Demszky, D. *et al.* (2020) 'GoEmotions : A Dataset of Fine-Grained Emotions', in *Proceedings of the 58th Annual Meeting of the Association for Computational Linguistics*, pp. 4040–4054.

Dzogang, F., Lansdall-Welfare, T. and Cristianini, N. (2016) 'Seasonal Fluctuations in Collective Mood Revealed by Wikipedia Searches and Twitter Posts', *IEEE International Conference on Data Mining Workshops, ICDMW*, 0, pp. 931–937. doi: 10.1109/ICDMW.2016.0136.

Dzogang, F., Lightman, S. and Cristianini, N. (2018) 'Diurnal variations of psychometric indicators in twitter content', *PLoS ONE*, 13(6). doi: 10.1371/journal.pone.0197002.

Ekkekakis, P. (2013) *The Measurement of Affect, Mood, and Emotion*, *Panteleimon Ekkekakis*. Cambridge University Press. doi: 10.1176/pn.39.2.0031b.

Ekman (1992) 'An argument for basic emotions', *Cognition and Emotion*, 6, pp. 169–200.

Entwistle, C., Marceau, E. and Boyd, R. L. (2022) 'Personality disorder and verbal behavior', *Handbook of language analysis in psychology.*, pp. 335–356. Available at: http://ovidsp.ovid.com/ovidweb.cgi?T=JS&PAGE=reference&D=psyc18&NEWS=N&AN=2022-08338-017.

Fineberg, S. K. *et al.* (2016) ‘Self-reference in psychosis and depression: A language marker of illness’, *Psychological Medicine*, 46(12), pp. 2605–2615. doi: 10.1017/S0033291716001215.

Furnham, A. (1983) ‘Social psychology as common sense’, *Bulletin of the British Psychological Society*, 36, pp. 105–109.

Golder, S. A. and Macy, M. W. (2011) ‘Diurnal and seasonal mood vary with work, sleep, and daylength across diverse cultures’, *Science*, 333(6051), pp. 1878–1881. doi: 10.1126/science.1202775.

Harvey, D. *et al.* (2022) ‘Natural Language Processing Methods and Bipolar Disorder: Scoping Review’, *JMIR Mental Health*, 9(4). doi: 10.2196/35928.

Ireland, M. and Iserman, M. (2018) ‘Within and Between-Person Differences in Language Used Across Anxiety Support and Neutral Reddit Communities’, in *Proceedings of the Fifth Workshop on Computational Linguistics and Clinical Psychology: From Keyboard to Clinic,* pp. 182–193. doi: 10.18653/v1/w18-0620.

Kahn, J. H. *et al.* (2007) ‘Measuring emotional expression with the Linguistic Inquiry and Word Count’, *American Journal of Psychology*, 120(2), pp. 263–286. doi: 10.2307/20445398.

Kennedy, B. *et al.* (2022) ‘Text Analysis for Psychology: Methods, Principles, and Practices’, *Guilford Press*, pp. 3–33.

Köper, M., Kim, E. and Klinger, R. (2017) ‘IMS at EmoInt-2017: Emotion Intensity Prediction with Affective Norms, Automatically Extended Resources and Deep Learning’, in *Proceedings of the 8th Workshop on Computational Approaches to Subjectivity, Sentiment and Social Media Analysis (WASSA)*, pp. 50–57. doi: 10.18653/v1/W17-5206.

Newell, E. E. *et al.* (2018) ‘You Sound So Down: Capturing Depressed Affect Through Depressed Language’, *Journal of Language and Social Psychology*, 37(4), pp. 451–474. doi: 10.1177/0261927X17731123.

O’Dea, B. *et al.* (2021) ‘The relationship between linguistic expression in blog content and symptoms of depression, anxiety, and suicidal thoughts: A longitudinal study’, *PLoS ONE*, 16(5 May), pp. 1–17. doi: 10.1371/journal.pone.0251787.

Park, A. and Conway, M. (2017) ‘Longitudinal changes in psychological states in online health community members: Understanding the long-term effects of participating in an online depression community’, *Journal of Medical Internet Research*, 19(3). doi: 10.2196/jmir.6826.

Pennebaker, J. W. *et al.* (2015) *The Development and Psychometric Properties of LIWC2015*. Austin. doi: 10.15781/T29G6Z.

Pennebaker, J. W. (2022) ‘Computer-Based Language Analysis as a Paradigm Shift’, *Handbook of language analysis in psychology*, pp. 576–587.

Pennebaker, J. W. and Beall, S. K. (1986) ‘Confronting a Traumatic Event. Toward an Understanding of Inhibition and Disease’, *Journal of Abnormal Psychology*, 95(3), pp. 274–281. doi: 10.1037/0021-843X.95.3.274.

Pérez, J. M., Giudici, J. C. and Luque, F. (2021) ‘pysentimiento: A Python Toolkit for Sentiment Analysis and SocialNLP tasks’, pp. 1–4. Available at: http://arxiv.org/abs/2106.09462.

R Core Team (2022) *R: A Language and Environment for Statistical Computing*. Vienna, Austria. Available at: https://www.r-project.org.

Sekulić, I. *et al.* (2018) ‘Not Just Depressed: Bipolar Disorder Prediction on Reddit’, in *9th Workshop on Computational Approaches to Subjectivity, Sentiment and Social Media Analysis (WASSA)*.

Brussels, pp. 72–78. doi: 10.18653/v1/P17.

Sekulic, I. and Strube, M. (2019) ‘Adapting Deep Learning Methods for Mental Health Prediction on Social Media’, pp. 322–327. doi: 10.18653/v1/d19-5542.

Stone, J. A. and Ryerson, N. C. (2022) ‘Tweeting about alcohol: Exploring differences in Twitter sentiment during the onset of the COVID-19 pandemic’, *Plos One*, 17(11), p. e0276863. doi: 10.1371/journal.pone.0276863.

Tackman, A. M. *et al.* (2018) ‘Depression, Negative Emotionality, and Self-Referential Language: A Multi-Lab, Multi-Measure, and Multi-Language-Task Research Synthesis’, *Journal of Personality and Social Psychology*, (March). doi: 10.1037/pspp0000187.

Tausczik, Y. R. and Pennebaker, J. W. (2010) ‘The psychological meaning of words: LIWC and computerized text analysis methods’, *Journal of Language and Social Psychology*, 29(1), pp. 24–54. doi: 10.1177/0261927X09351676.

Tay, D. and Qiu, H. (2022) ‘Modeling Linguistic (A)Synchrony: A Case Study of Therapist–Client Interaction’, *Frontiers in Psychology*, 13(May). doi: 10.3389/fpsyg.2022.903227.
