# Supplementary material for: Posting patterns in peer online support forums and their associations with emotions and mood in bipolar disorder: Exploratory analysis
Source: PLoS One. 2023 Sep 25;18(9):e0291369. doi: 10.1371/journal.pone.0291369 (PMC10519601; doi:10.1371/journal.pone.0291369)
Supplement: Attachment — Submitted filename: BipolarPostingMood_PLOS_R2_ReviewerResponse.docx [file pone.0291369.s009.docx]

**PONE-D-22-26677**

**Posting patterns in peer online support forums and their associations with emotions and mood in bipolar disorder: Exploratory analysis**

**PLOS ONE**

# Response to Reviewers

For ease of checking the revisions, we have added the reviewer and comment number to all changes in the Track Changes version of the revised manuscript and copied them in this response letter.

## Reviewer 3 comments to the authors

**R3.1** This is an interesting study. Bringing in lived experience was a nice touch.

**Our response:**

Thank you very much for your appreciation of our study and recognising the value of lived experience in research.

**R3.2** I do, however, have a few suggestions. One is that people may seek out forums like Reddit because they feel more comfortable even if they do have access to health care.

**Our response:**

Thanks for this suggestion. We agree and have added this aspect to the introduction. **Our changes:**

In light of the global Covid-19 pandemic, where in-person care has become more difficult to access for many people, international BD experts have highlighted the importance of online MH support and the “great need” to “train and support clinicians to go where people are already online” [25]. People may also use online forums in addition to in-person MH care since quantitative [26,27] and qualitative [28,29] evidence shows that online anonymity affords personal self-disclosures and discussions of sensitive and stigmatised issues.

**R3.3** I would urge you to go over the introduction again because it seems to be redundant in places

**Our response:**

We have edited the introduction as recommended, also taking up some suggestions of reviewer 4.

**Our changes:**
The internet, including social media and online support forums, plays an increasingly important role in sharing information and support for health and mental health (MH) concerns [18,19]. Like the general population, most people with a (BD) diagnosis are internet users [20,21] and seek information about BD online [22]. In 2014-15, 13%-17% of people with a BD, major depressive disorder, or psychotic disorder diagnosis reported posting and seeking information on their condition in online support forums or social media [23,24]. Since then, the importance of online MH forums has likely increased. The online discussion platform Reddit (reddit.com) is one of the most visited internet sites worldwide and hosts subforums (subreddits) for a variety of topics, ranging from general interests to specific topics, including MH [39]. In 2019, the number of members in ~~support forums for BD on the online discussion platform Reddit~~ BD subreddits (bipolar, BipolarReddit, bipolar2, bipolarSOs) increased by more than 50%. In light of the global Covid-19 pandemic, where in-person care has become more difficult to access for many people, international BD experts have highlighted the importance of online MH support and the “great need” to “train and support clinicians to go where people are already online” [25]. People may also use online forums in addition to in-person MH care since quantitative [26,27] and qualitative [28,29] evidence shows that online anonymity affords personal self-disclosures and discussions of sensitive and stigmatised issues.

There are a number of content analyses of BD online forums [30–36], including ~~Reddit forums~~ subreddits [37]. Jagfeld, Lobban, Rayson, et al. [38] characterised the demographics (age, gender identity, country of origin, comorbidities) of Reddit users with a self-reported BD diagnosis. However, little is known about the users of online MH support forums and why some users chose to engage in them and others not. This knowledge is both relevant to improve online MH support forum design and to contextualise the increasing body of research relating to such forums.

~~The online discussion platform Reddit (reddit.com) is one of the most visited internet sites worldwide and hosts subforums (subreddits) for a variety of topics, ranging from general interests to specific topics, including MH [39]. Researchers increasingly analyse Reddit data [40,41] and it is one of the main sources of data for research on BD with natural language processing (NLP) methods [42].~~

**R3.4** I found the description of the information in Table 3 difficult to understand. Your readers might not always be sophisticated statistically, particularly in the case of mental health.

**Our response:**
Thank you very much for pointing this out. We have added a reference to the method section that explains how to compare the fit of linear mixed-effects models and a key to the table.

**Our changes:**
Model fit for the model with only the control variables vs. control and LIWC variables was compared via a likelihood ratio test with X^2^ statistics, following best practice guidance for linear mixed-effects models in psychological science (Meteyard and Davies, 2020).

Table key: LRT: likelihood ratio test, AIC: Akaike Information Criterion, BIC: Bayesian Information Criterion, LL: log likelihood, df: degrees of freedom, X2: Analysis of deviance (deviance $X^{2}$, $P(>X^{2})$)

**R3.5** Finally, it would be great if you had some ideas about how to help participants cope better

**Our response:**

As stated in the future work part of the discussion, the people with lived experience of BD that we talked to, particularly wished for recommendations how to use social media and online support forums to achieve good long-term quality of life or personal recovery. The Improving Peer Online Forums study, which aims to find out how online MH forums work, why some work better than others, and why some people find them helpful and others do not, may yield promising evidence for this. However, it is still ongoing, so there are no recommendations to share yet.

## Reviewer 4 comments to the authors

**Our response:**

Thank you for your detailed comments on our manuscript. We have done our best to address them as indicated in our responses below.

**R4.1** The methods are hard to read. The presence of subsections is confusing and does not allow prompt overall comprehension. In addition, the inclusion and exclusion criteria are not easily accessible; some are even in the results section. I suggest the authors rewrite this section considering a different sub-section scheme: i) inclusion and exclusion criteria (including self-report diagnosis, comorbidities, number of posts, karma, user age when posting, etc.); ii) Reddit user identification, mental health subreddit identification (also, the ‘line 165’ includes results (quantitative data) that should be moved to the appropriate section); iii) language analysis (well-written btw); iv) outcomes (research questions); v) ethical considerations; vi) involvement of people with lived experience (herein some sentences (e.g., lines 284-291), are results (even if narrative) and should not be presented in methods.

**Our response:**

Thank you for your suggestions on how to make this section more reader friendly.
We brought together the inclusion and exclusion criteria in the section ‘Reddit user identification and inclusion and exclusion criteria’ (previously ‘Reddit user identification and dataset construction’). The statistical analyses for the research questions are now detailed in the section ‘Statistical analyses’ rather than in individual subsections. We also moved line 165 to the results section.

You remark that lines 284-291 in the subsection ‘Involvement of people with lived experience’ are results and should not be presented in the methods section. The volunteers gave this feedback during the preparation of this study and it informed our expectations for the research questions. Therefore, we think it is important to state this prior to presenting the results to keep our preparatory PPI consultation separate from the research findings. The ‘Feedback from people with lived experience’ subsection in the results section presents the volunteers’ feedback on the actual study results.

**Our changes:**
Reddit user identification and ~~dataset construction~~ inclusion and exclusion criteria

Reddit users who stated on the platform that they had received a BD diagnosis from a professional were automatically identified via self-reported diagnosis statements like ‘I was diagnosed with bipolar disorder’ [see 39]. All posts of candidate users (id, text, timestamp created at, subreddit) between 01/2005 (site inception) and 03/2019 (data available at time of data collection in 05/2019) were downloaded via the RedditAPI praw [57]. Candidate users were filtered by matching all of their posts against 74 exclusion patterns like ‘not officially diagnosed’ or ‘self-diagnosed’. Also, users who reported a psychotic disorder diagnosis were removed, as this constitutes an exclusion criterion for BD according to DSM-5 [1]. Users with other MH comorbidities, including major depressive disorder, were retained. ~~The final Self-reported BD diagnosis (S-BiDD) dataset comprises 21,407,595 posts (both submissions (thread starts) and comments) of 19,685 users with a self-reported BD diagnosis.~~

RQ2 only concerned a subset of users with at least four posts of at least 25 words each in both MH and non-MH subreddits. Comparable studies of Reddit language using LIWC required at least four posts in depression subreddits [54] or five posts overall [82]. To address RQ3, these users who had posted in MH subreddits were compared against users who had no posts in MH subreddits and at least eight posts with at least 25 words each in non-MH subreddits. Users for which the NLP methods could not assign the control variables age or gender (see Section ‘Statistical analyses’ below) were excluded in the analyses to address RQ3.

Statistical analyses

~~RQ 1: What proportion of Reddit users with a BD diagnosis posts in MH and non-MH subreddits?~~

To address RQ1, the percentages of users who posted only in MH subreddits, only in non-MH subreddits or in both were calculated. Previous work considered only Reddit users with at least four posts in MH subreddits as active contributors and those with fewer posts as lurkers [54]. Therefore, the number of users with at least four posts in both MH and non-MH subreddits was also calculated, and, for comparison, the number of users with at least eight posts who posted only in MH or non-MH subreddits.

~~RQ 2: What differences exist in the emotions that Reddit users with a BD diagnosis express in MH and non-MH subreddit posts?~~

To address RQ 2, the mean LIWC variable scores of the posts of users in MH subreddits were compared to the mean scores in non-MH subreddits via dependent t-tests with Cohen's d as the effect size measure [84]. ~~Only users with at least four posts with at least 25 words each in both MH and non-MH subreddits were included. Comparable studies of Reddit language using LIWC required at least four posts in depression subreddits [54] or five posts overall [82].~~

~~RQ 3: How does mood differ between users who post in MH subreddits and those who only post in non-MH subreddits?~~

To address RQ3, a logistic regression model was fitted via the R [85, Windows version 4.1.0] glm function to predict whether a user had posted in MH subreddits based on the mean LIWC variable scores of their non-MH subreddit posts and the control variables described below. Model fit for the model including only the control variables versus the model including control and LIWC variables was compared using a likelihood ratio test, following best practice guidance for linear mixed-effects models in psychological science [102]. See S2 Appendix for method details. The outcome variable *posted in MH* was coded as 1 for users who had ~~at least four posts~~ posted in MH subreddits and ~~at least four posts in non-MH subreddits with at least 25 words each (same users as in RQ 2), and~~ 0 for ~~users who had no posts in MH subreddits and at least eight posts with at least 25 words each in non-MH subreddits~~ those who had not (see ‘Reddit user identification and inclusion and exclusion criteria’). Importantly, only non-MH subreddit posts were used to measure mood to provide a more comparable source of measures for users from both groups. LIWC emotion variables might be affected by the communicative context [86]. Only including posts made in non-MH subreddits avoided conflating mood and context.

**R4.2** Also, there is no indication of employed data analysis (e.g., cohen’s d, OR, chi-square). They should be stated and added as a sub-section of methods.

**Our response:**

We have added the analysis measures that had previously not been stated in the methods section.

**Our changes:**

To address RQ 2, the mean LIWC variable scores of the posts of users in MH subreddits were compared to the mean scores in non-MH subreddits via dependent t-tests with Cohen's d as the effect size measure (Cohen, 1988).

To address RQ3, a logistic regression model was fitted via the R [85, Windows version 4.1.0] glm function to predict whether a user had posted in MH subreddits based on the mean LIWC variable scores of their non-MH subreddit posts and some the control variables described below. Model fit for the model with only the control variables vs. control and LIWC variables was compared using a likelihood ratio test, following best practice guidance for linear mixed-effects models in psychological science [102]. See S2 Appendix for method details.

**R4.3** The sequence of the sub-sections is indicative and not mandatory at all.

**Our response:**

We think the sequence of sub-sections is sensible and have followed it.

**R4.4** I do not really like the RQ presentation. I would rephrase them in ‘outcomes’ to be presented in a separate subsection and move the scattered inclusion criteria to a different section.

**Our response:**
Thanks for this suggestion. We have decided to keep the RQ presentation since we have structured the whole paper around this. However, following your advice, we collated the statistical analyses for the three RQs in one method subsection called ‘Statistical analyses’. We also brought together the inclusion criteria in the new section ‘Reddit user identification and inclusion and exclusion criteria’.

**Our changes:**
See changes for R4.1

**R4.5** How did you handle the [deleted] users, given that you included accounts with <4 posts? Did the program automatically exclude them?

**Our response:**

Yes, that is correct. For each research question we selected only the users from the S-BiDD dataset that fulfilled the eligibility criteria.

**R4.6** 2. Line 236: ‘Some control variables’: what are we talking about? Is this presented in methods?

**Our response:**

We clarified that these are the control variables presented in this subsection below.

**Our change:**

To address RQ3, a logistic regression model was fitted via the R (R Core Team, 2022, Windows version 4.1.0) glm function to predict whether a user had posted in MH subreddits based on the mean LIWC variable scores of their non-MH subreddit posts and ~~some~~ the control variables described below.

**R4.7** 3. Line 239: why not >4 posts like before? By the way, this should be moved in inclusion criteria in methods.

**Our response:**

We required users with *posted in MH* = 0 to have at least 8 posts because users with *posted in MH* = 1 had to have at least eight posts (at least four in MH and non-MH subreddits each). We wanted to keep the minimum number of posts the same in both outcome groups to rule out the possibility that any differences we would find would be due to users posting more frequently in the *posted in MH* = 1 group.

**R4.8** 4. Line 307: no, you did not write the eligibility criteria.

**Our response:**

We previously stated the eligibility criteria as part of the methods subsection for RQ2. Following your suggestion, we now include them in the method subsection titled ‘Reddit user identification and inclusion and exclusion criteria’.

**Our change:**

N=9,821 users (49.9% of total) with 6,493,626 posts of at least 25 words (11.4% in MH subreddits) met the ~~eligibility~~ criteria set out in the ‘Reddit user identification and inclusion and exclusion criteria’ section as active contributors in both MH and non-MH subreddits with at least four posts in each.

**R4.9** 5. Table 2: anger effect size interpretation is ‘no effect’, but the p-value is <.001, please check all p-values.

**Our response:**
Thank you very much for being so attentive and spotting this. We use the categories proposed by Sawilowsky (Sawilowsky, 2009) (d (.01)=very small, d (.2)=small, d (.5)=medium, d (.8)=large, d (1.2)=very large, and d (2.0)=huge) and have added the reference to the table to make this clear. Upon checking this, we found that the interpretation of the effect for anger should indeed be ‘Very small’ instead of ‘No effect’. We have also corrected the effect size interpretation of ‘age’ in S2 Table from ‘No effect’ to ‘Very small’ and checked all other effect size interpretations.

Our changes:
There is a significant difference between users’ posts in MH and non-MH subreddits for each preselected LIWC variable ~~except anger~~ (Table 2).

Table 2. Within user differences between MH and non-MH subreddit posts.

|  | MH posts | Non-MH posts | Dependent t-test, *** = p < 0.001 with Bonferroni correction for 5 comparisons |
| --- | --- | --- | --- |

|  | mean | std | mean | std | *P* | effect size (Cohen's d) | effect size interpretation (Sawilowsky, 2009) |
| --- | --- | --- | --- | --- | --- | --- | --- |
| positive emotion | 3.48 | 1.08 | 3.64 | 0.93 | <.001 | -0.20 | Small |
| anxiety | 0.67 | 0.42 | 0.37 | 0.22 | <.001 | 0.97 | Large |
| anger | 0.75 | 0.49 | 0.78 | 0.44 | <.001 | -0.07 | Very small |
| sadness | 0.81 | 0.44 | 0.45 | 0.24 | <.001 | 1.11 | Large |
| 1^st^ pers. sg. | 8.82 | 2.28 | 6.39 | 1.96 | <.001 | 1.63 | Very large |

S2 Table. Descriptive statistics and between user differences for users in the regression model.

|  | Posted in MH subreddits | | Never posted in MH subreddits | | a: Independent t-test; b: Welch’s t-test | | |
| --- | --- | --- | --- | --- | --- | --- | --- |
|  | mean | std | mean | std | *P* | effect size a: Cohen's d  b: Glass’ delta | effect size interpretation  (Sawilowsky, 2009) |
| age | 29.22 | 9.31 | 29.47 | 9.82 | 2.28^a^ | -0.03^a^ | Very small |

**R4.10** 6. Table 4: active days. 95%C.I. <.001, <.001, as well as the p-value. Please double-check.

**Our response:**

The exact values for the 95% CI for active days are 0.0003 – 0.0004 (please note that these are coefficient estimates and not p-values). We have decided to display them as <.001– <.001 for the sake of consistently reporting only two decimals. We double-checked the p-value and it is indeed < 2e-16.

**R4.11** 7. Results: Please consider moving line 165 at the start of the results, along with the results summary. Before the sub-sections, you can write Fig. 2 provides a visual summary of all results.

**Our response:**
Thank you for this suggestion, we have taken it up (see changed start of the results section below).
 **Our changes:**

**Results**

**Results summary**

The final Self-reported BD diagnosis (S-BiDD) dataset comprises 21,407,595 posts (both submissions (thread starts) and comments) of 19,685 users with a self-reported BD diagnosis. Fig. 2 provides a visual summary of all results. Two thirds of the identified 19,685 users with a self-reported BD diagnosis posted in both MH and non-MH subreddits, while 24% never posted in MH subreddits. Users who posted in both MH and non-MH subreddits used more first-person singular pronouns and exhibited more sadness and anxiety, but less positive emotion in their MH subreddit posts. Feminine gender and expressing more anxiety, sadness, and positive emotion in non-MH subreddit posts were significant predictors for posting in MH subreddits. The following subsections present the detailed results for the three research questions.

**Fig. 2. Visual summary of the results.**

**R4.12** Also, the RQ style could be rephrased to get outcome-like headings.

**Our response:**

Thanks for this suggestion. After careful consideration, we have decided to keep the RQ presentation.

**R4.13** A style suggestion: when you state ‘Fig.1 shows, table 2 shows’, you may rephrase the sentence and put (Table 1) or (Fig.2) at the end.

**Our response:**

Thanks for this suggestion, we have edited some cross references accordingly.

**Our changes:**

Two thirds of the 19,685 identified Reddit users who disclosed a BD diagnosis posted in both MH and non-MH subreddits (Fig. 1).

There is a significant difference between users’ posts in MH and non-MH subreddits for each preselected LIWC variable (see Table 2).

The regression model including the LIWC variables as predictors fit the data better than the model with the control variables only (see Table 3).

**R4.14** 8. Discussion: lines 487-490 should be the first sentences of the discussion.

**Our response:**

We have edited the start of the discussion as requested.

**Our changes:**

See changes for R4.15.

**R4.15** Remove the ‘principal results’ heading and include lines 358-360 to 487-490.

**Our response:**
We have edited the start of the discussion as requested, also including your suggestion R4.21.

**Our changes:**

Discussion

~~Principal Results~~

~~By applying psychological text analysis to a large sample of Reddit users with a self-reported BD diagnosis,~~ This ~~exploratory~~ study shed more light on the posting behaviour of Reddit users with a self-reported BD diagnosis ~~these users~~ and the provides the first quantitative evidence on ~~associations between posting and emotions~~ the relationship between mood and posting in BD support forums. A strength of this study was the automatic user identification via self-reported diagnosis statements, which resulted in a large sample of participants. Since the data collection did not require direct interaction with the social media users, there was no researcher influence on the behaviour of the participants. Moreover, the psychological text analysis tool LIWC provided easily interpretable measures for in-the-moment emotion assessments, unbiased by self-report. Its wide use facilitated comparison with related work and its psychometric validity to measure emotions has been well established [68]. However, due to a lack of previous research, the study was necessarily exploratory. Future research should be carried out to confirm the findings and explore possible causal relationships between posting behaviour and mood.

**R4.16** Lines 437-438 are redundant.

**Our response:**

We have removed the introductory line of the implication sections as requested.

**Our changes:
Implications**

~~The findings of this study have important implications for research and practice.~~
**Implications for research.**

**R4.17** Lines 451-453 should be discussed earlier because they talk about a general result.

**Our response:**

We already discuss this finding in the discussion section for RQ 3 (‘Another finding was that users with a feminine gender identity were 33% more likely to post in MH subreddits compared to users with a masculine gender identity. […]’). Therefore, we did not make a change to the manuscript.

**R4.18** You can make this section more reader-friendly by removing the RQ subsections and trying to discuss the results by dividing the outcomes with a simple carriage return.

**Our response:**

Thank you very much for this suggestion. However, we have decided to keep the subheadings to keep a consistent structure between the methods, results, and discussion section. Furthermore, subheadings allow the readers to automatically jump to the research question that interests them.

**R4.19** Lines 442-449 are a staccato of the RQ2 discussion (earlier stated).

**Our response:**

Thanks for pointing this out. We have removed the corresponding paragraph from the RQ2 discussion.

**Our change:**
~~Overall, the findings demonstrate substantial differences in LIWC scores between posts in MH and non-MH contexts. Although this could be expected, this had not been empirically tested so far. This provides strong support for comparative language analyses and detection systems of people with and without MH issues to only consider posts in a comparable context as done in more recent research [44–46], compared to earlier work [42,43].~~

**R4.20** The ‘implications for practice’ sub-section is fine.

**Our response:**

Thank you.

**R4.21** The section ‘strengths, limitations, and future work’ should be changed to ‘limitations of the study’, and the ‘strengths of the study’ should be presented at the start of the discussion.

**Our response:**

We have edited the section as requested and present only limitations and future work.

**Our changes:**
See also changes for R4.15.

~~Strengths,~~ Limitations and future work

This study has three limitations that future research could address. ~~First, the automatic user identification via self-reported diagnosis statements resulted in a large sample of participants. Since the data collection did not require direct interaction with the social media users, there was no researcher influence on the behaviour of the participants. However,~~ It was not possible to check the veracity of self-reported diagnoses, although it appears unlikely that people would deliberately unfaithfully identify with a stigmatised MH condition. Cluss et al. [111] verified that 93% of 804 individuals who self-reported BD diagnosis met criteria for a lifetime BD diagnosis according to an in-person structured diagnostic interview. Nevertheless, future complementary research could conduct diagnostic interviews to determine the eligibility of participants, which would also allow inclusion of people who meet criteria for BD but have not received a diagnosis yet.

~~Second, the text analysis tool LIWC provided easily interpretable measures for in-the-moment emotion assessments, unbiased by self-report. Its wide use facilitated comparison with related work and its psychometric validity to measure emotions has been well established [68].~~ LIWC is only one tool to study the mood of social media users. Future work could triangulate Lthe present results with emotion text analysis systems trained via machine learning that may be more accurate in context but less interpretable than the dictionary-based LIWC tool [79], as well as with self-reported ~~mood~~ questionnaires and qualitative interviews. ~~Third, this study is the first to provide quantitative evidence of the relationship between mood and engaging with MH support forums for BD. Due to a lack of previous research, the study needed to be exploratory and future research should be carried out to confirm the findings and explore possible causal relationships between posting behaviour and mood. Future research should also explore whether the present findings are specific to BD or mood or MH issues more general.~~ Finally, this study only considered data until March 2019. While online MH forum usage has likely increased since then, fuelled by the global Covid-19 pandemic [112], there are no apparent reasons why the usage patterns found here would have changed. However, future research could replicate these analyses with more recent data and consider changes over time. Future research should also explore whether the present findings are specific to BD or mood or MH issues more general.

**R4.22** Try avoiding stating first, second, and third.

**Our response:**

We have removed the enumeration in the limitations section as requested.

**Our changes:**

See changes for R4.21.

**R4.23** 9. Abbreviations: it is redundant to present abbreviations in a separate section. You can write the (abbreviation) the first time you use a phrase, then write the abbreviation. Watch out, MH is already present at line 68, while ‘mental health’ is stated at line 72 for the first time. Double-check all other abbreviations.

**Our response:**

We have removed the Abbreviations section. We moved the introduction of the MH abbreviation to the first mention and checked all other abbreviations as suggested.

**R4.24** 10. Miscellaneous: Line 61: ‘extreme high and low mood states’ is not really a good way to describe the core criteria of bipolar disorder diagnosis. Consider rephrasing it.

**Our response:**

We have rephrased the core criteria of a bipolar disorder diagnosis, now directly quoting [ICD-11](https://icd.who.int/browse11/l-m/en#/http://id.who.int/icd/entity/613065957): ‘Bipolar and related disorders are episodic mood disorders defined by the occurrence of Manic, Mixed or Hypomanic episodes or symptoms. These episodes typically alternate over the course of these disorders with Depressive episodes or periods of depressive symptoms.’

**Our changes:**

Experiences of ‘Manic, Mixed, or Hypomanic episodes or symptoms’ alternating with ‘Depressive episodes or periods of depressive symptoms’ that interfere with functioning, constitute core criteria for a bipolar disorder (BD) diagnosis [1,2].

**R4.25** Lines 63-64 content is redundant (you state the countries in brackets, then state them again).

**Our response:**
The cited studies include data from 22 different countries, we only stated the extremes and an in-between value.

**Our changes:**

Prevalence rate estimates for bipolar spectrum disorders range from 0.1% (India), 2% (England), to 4.4% (US) across 22 different European, American, and Asian countries [3–5].

**R4.26** Lines 89-91 (until [35]) should go before line 83, and the two paragraphs should merge and harmonize.

**Our response:**

Thanks for this suggestion. We have moved lines 89-91 even earlier to remove some redundancy in the introduction and merged the two paragraphs as suggested.

**Our changes:**

See changes for R3.3.

**R4.27** Line 130: what does ‘emotional tone improves’ mean? I struggle to believe that own’s emotional tone improves while talking in the suicidality subreddit.

**Our response:**
Indeed, this is what Silveira et al. found for the four subreddits they looked at. The emotional tone of a thread initiator’s last comment was usually more positive than the post that they started the thread with (see (Silveira *et al.*, 2021, fig. 2). We have expanded the sentence to clarify this.

**Our change:**

Text emotion analysis with machine learning methods [53] has shown that users’ emotional tone improves during exchanges in four large MH subreddits for depression, suicidality, BD, and anxiety when comparing their initial submission to their last comment in the same thread.

**R4.28** Line 164: bipolar disorder automatically excludes the possibility of a ‘depression’ diagnosis (you talk about DSM-5, then write depression) or, better, a major depressive disorder. The only chance is a major depressive episode in a bipolar patient. Please rephrase.

**Our response:**

Thank you for pointing this out. We have replaced ‘depression’ with ‘major depressive disorder’ and clarified our reasoning for retaining users who also reported this diagnosis.

**Our changes:**
Candidate users were filtered by matching all of their posts against 74 exclusion patterns like ‘not officially diagnosed’ or ‘self-diagnosed’. All posts of these users were also matched against self-reported diagnosis statements for nine other MH diagnoses [39]. ~~Also, users who reported a~~ A major depressive disorder or psychotic disorder diagnosis ~~were removed, as this~~ constitutes an exclusion criterion for BD according to DSM-5 [1]. However, around 40% of people who are diagnosed with BD first received a major depressive disorder diagnosis [58,59]. Therefore, only users who also reported a psychotic disorder diagnosis were removed. ~~Users with other MH comorbidities, including depression, were retained.~~

## References

Cohen, J. (1988) *Statistical power analysis for the behavioral sciences*. 2nd edn. Erlbaum.

Meteyard, L. and Davies, R. A. I. (2020) ‘Best practice guidance for linear mixed-effects models in psychological science’, *Journal of Memory and Language*, 112(March 2019), p. 104092. doi: 10.1016/j.jml.2020.104092.

R Core Team (2022) *R: A Language and Environment for Statistical Computing*. Vienna, Austria. Available at: https://www.r-project.org.

Sawilowsky, S. S. (2009) ‘New Effect Size Rules of Thumb’, *Journal of Modern Applied Statistical Methods*, 8(2), pp. 597–599. doi: 10.22237/jmasm/1257035100.

Silveira, B. *et al.* (2021) ‘Predicting user emotional tone in mental disorder online communities’, *Future Generation Computer Systems*, 125, pp. 641–651. doi: 10.1016/j.future.2021.07.014.
